# Rheological and Drug Delivery Characteristics of Poloxamer-Based Diclofenac Sodium Formulations for Chronic Wound Site Analgesia

**DOI:** 10.3390/pharmaceutics12121214

**Published:** 2020-12-15

**Authors:** Jackson Russo, Jennifer Fiegel, Nicole K. Brogden

**Affiliations:** 1 Department of Pharmaceutical Sciences and Experimental Therapeutics, The University of Iowa, Iowa City, IA 52242, USA; jackson-russo@uiowa.edu; 2 Department of Chemical and Biochemical Engineering, The University of Iowa, Iowa City, IA 52242, USA; jennifer-fiegel@uiowa.edu; 3 Department of Dermatology, The University of Iowa, Iowa City, IA 52242, USA

**Keywords:** topical, poloxamer, thermogelation, chronic wounds, analgesia

## Abstract

Chronic wounds are a significant and growing health problem, and clinical treatment is often a painful experience. A topical dosage form would be optimal to treat this pain. Poloxamer 407, a thermosensitive polymer that is a liquid at low temperatures but gels at higher temperatures, is well suited to administer topical analgesics to chronic wound sites. The goal of this study was to evaluate the gelation and drug delivery properties of poloxamer 407 gels containing diclofenac sodium for potential use in chronic wound analgesic delivery. The gelation properties of poloxamer formulations were evaluated rheologically. Drug delivery properties of poloxamers loaded with diclofenac sodium were evaluated using snakeskin dialysis membranes, intact porcine ear skin, and porcine ear skin impaired via tape stripping. A commercial gel product and a solution of diclofenac sodium in water were used as control formulations. Poloxamer concentration and gelation temperature varied inversely, and the addition of higher concentrations of diclofenac sodium correlated to significant increases in poloxamer gelation temperature. Poloxamer solutions were effective in limiting the permeation of diclofenac sodium through membranes with impaired barrier properties, and delivery of diclofenac sodium from poloxamer 407 did not vary significantly from delivery observed from the commercial gel product. The amount of drug delivered in 24 h did not change significantly with changes in poloxamer 407 concentration. The results of this study indicate that poloxamer 407 may be a useful formulation component for administration of an analgesic product to a chronic wound site.

## 1. Introduction

The skin is the largest organ of the body and is key to maintaining homeostasis, with a unique layered structure that provides a barrier to harmful external materials and maintains body temperature and moisture [1,2,3]. However, as the body’s most exposed organ, the skin is also prone to external insults that can create open wounds and compromise its critical homeostatic functions. While most wounds heal in a timely manner, around 6.5 million Americans annually suffer from chronic wounds that undergo a greatly prolonged healing process [4].

Pain management correlates with shortened wound healing times and improved patient outcomes and thus is key to improving the quality of life for patients suffering from chronic wounds [5,6,7,8]. Systemic analgesic compounds such as orally administered non-steroidal anti-inflammatory drugs (NSAIDs) and opioids can provide pain relief. However, ongoing administration of these compounds over the duration of a prolonged treatment course can lead to adverse effects. Prolonged oral administration of NSAIDs is associated with peptic ulcer formation and renal failure, particularly in elderly patients. Long-term administration of systemic opioids is associated with constipation, respiratory depression, and potential for addiction and misuse [9,10].

A topical analgesic product would limit systemic exposure and mitigate systemic adverse effects while providing localized pain management in the wound. However, the administration of a topical product is likely to involve prolonged wound site contact, which could increase the pain involved in wound dressing changes [11,12]. Administration of a therapeutic compound directly into a wound can also result in rapid absorption of the drug due to the lack of a skin barrier, which would diminish local analgesic efficacy [13]. In order to reduce the frequency of analgesic reapplication and dressing changes, and limit rapid systemic absorption, it is important that a drug depot source is provided to offer prolonged local delivery of the analgesic compound. A product that can be applied with minimal contact to the wound site itself while providing a slow-release depot of an analgesic drug would therefore be a significant advance in the optimization of chronic wound pain management.

**Figure 1:**
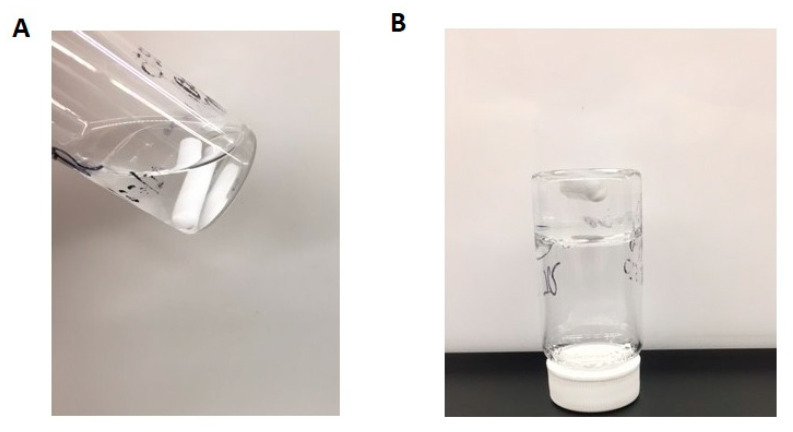
Solution of 20% *w/w* poloxamer 407 in water (**A**) at 4 °C prior to heating and (**B**) after heating on a stir plate until gelation occurred, causing the stir bar to stop moving.

Thermosensitive polymers that undergo reverse thermal gelation are well suited to meet the unique challenges presented by chronic wounds. Reverse thermal gelation is the process by which a polymer transitions from a liquid state at low temperatures to a gel state when heated [14]—the liquid to gel transition of poloxamer 407 is shown in Figure 1. Polymers that undergo reverse thermal gelation could be administered as cool liquids with minimal wound site contact, and a gel would form within the wound site to provide a local depot source of the analgesic. Poloxamer 407 is a thermosensitive polymer vehicle that has low toxicity, lack of skin irritation, and reverse thermal gelation properties. In addition, poloxamer 407 systems containing a variety of drug compounds, including small-molecule drugs and proteins, have been shown to remain stable as solutions when stored at 4 °C for up to 3 months [15,16]. It has been studied for use in transdermal delivery, often in combination with either permeation enhancers or microneedles, to overcome the barrier properties of the skin [17]. However, poloxamer 407 is similarly promising for use in the delivery of analgesics to chronic wounds by prolonging release of a topically administered analgesic compound to a site that does not have an intact skin barrier.

Past studies have been conducted exploring thermosensitive polymers as potential formulation ingredients to deliver analgesic compounds, but the delivery of an analgesic compound topically to a chronic wound site has not yet been adequately explored. A series of studies was conducted to explore poloxamer 407 as an ingredient in a suppository formulation for the prolonged systemic delivery of diclofenac sodium [18]. Additionally, poloxamer 407 has been studied for use in a vehicle for local injection of a prolonged dose of lidocaine hydrochloride [19]. Poloxamer 407 has also been studied in the past as a vehicle to prolong the delivery of morphine to large skin wounds. However, the model membranes used in the drug delivery study were designed to mimic the characteristics of full-thickness skin, and there were limited data presented that would indicate the effect of administration of the analgesic compound in a poloxamer 407 vehicle to an impaired skin site [20]. Wound sites lack the barrier characteristics of intact skin, which allows drug compounds administered to wound sites to diffuse more rapidly into systemic circulation and out of the intended area of effect than drug compounds administered to full-thickness skin [13]. Therefore, studies using impaired skin membranes are key in determining whether poloxamer 407 has the potential to be useful as a clinically relevant vehicle for prolonged topical delivery of analgesic drugs to chronic wound sites. Although it has been studied in the past for the delivery of analgesic compounds, the potential of poloxamer 407 as a means of prolonging drug presence in impaired skin and specifically in the environment of a chronic wound site has not yet been adequately explored.

Diclofenac sodium, an NSAID approved for use in topical analgesia, was chosen as a model analgesic compound for these studies. It would be advisable for an eventual product developed for use in chronic wound analgesia to contain multiple analgesic compounds to address both the acute pain response mediated by prolonged inflammation of the chronic wound site and the chronic pain caused by deeper nerve damage [21]. However, diclofenac sodium is a convenient analgesic candidate to use in early drug delivery studies, offering well-developed quantification methods and an array of commercial products for comparison. Additionally, although NSAIDs have been shown to prolong the healing process in acute wounds, there is potential for them to be useful in chronic wound care [22]. The delayed mechanism of healing in chronic wounds is frequently due to their suspension in the inflammatory phase of the wound cycle. This leads to an overabundance of compounds designed to break down the damaged extracellular membrane of the wound site, which prevents fibroblast accumulation within the wound site and ultimately prevents progression to the proliferative phase [23]. There are indications that locally acting anti-inflammatory drugs may help chronic wounds to progress past the inflammatory phase by relieving this excessive inflammation [24,25,26]. Therefore, in addition to being a convenient model analgesic compound, the anti-inflammatory properties of diclofenac sodium could be well suited to expediting the healing process of chronic wounds.

In this research, we evaluated poloxamer 407 as a potential vehicle for the delivery of the model analgesic compound, diclofenac sodium. The goals of the current studies were to (1) rheologically study poloxamer 407 solutions for in vitro gelation characteristics both with and without diclofenac sodium and (2) measure drug release and delivery characteristics when applied to excised skin with either an intact or an impaired barrier.

## 2. Materials and Methods

Poloxamer 407 was purchased from Anatrace (Maumee, OH; sold as Pluronic^®^ F-127). Diclofenac sodium salt, gentamicin sulfate, HPLC-grade water, and acetonitrile were purchased from Sigma-Aldrich (St. Louis, MO, USA). Formic acid was purchased from Avantor (Center Valley, PA, USA). HEPES free acid and solid sodium bicarbonate were purchased from Research Products International (Mt. Prospect, IL, USA). Sodium hydroxide solution (1 N) was purchased from Fisher Chemical (Fair Lawn, NJ, USA).

### 2.1. Preparation of Poloxamer Gels

Poloxamer gels containing no active pharmaceutical ingredient were made as 17% and 20% *w/w* solutions. The gels were prepared via slow dissolution of solid poloxamer 407 in deionized water under constant stirring overnight at 4 °C. To prepare gels with a final diclofenac sodium concentration of 0.5%, 1%, 1.5%, and 2% *w/v*, diclofenac sodium solid was added to the 17% and 20% *w/w* poloxamer solutions on the day of the experiment under cold stirring for approximately 1 h until dissolved. All gels were stored at 4 °C until use.

### 2.2. Rheologic Characterization of Poloxamer Gels

An ARES-G2 rotational rheometer (TA Instruments, New Castle, DE, USA) was used to determine poloxamer gelation characteristics. First, the linear viscoelastic regions (LVR) of the poloxamer gels were determined in order to select an appropriate oscillation stress at which to conduct further rheological measurements. The LVR is the range of applied strain for which the viscoelastic character of a material, as reflected by its storage modulus, remains unchanged [27]. To find the LVR, the poloxamer 407 gels were positioned within a 1 mm gap between 50 mm parallel plates and allowed to equilibrate at a temperature of 37 °C for 5 min to ensure that measurements were being performed on the gelled sample. After equilibration, the storage modulus was measured as the gels were subjected to oscillation strain that steadily increased from 0.1% to 100% at an oscillation frequency of 6.28 rad/s. The LVR was reported as the range of oscillatory strain for which the observed storage modulus of the material changed by less than 5% with subsequent measurement. Sample temperature was precisely controlled using a Peltier Plate attachment on the instrument, and solvent evaporation was prevented by using a solvent trap.

Changes in the storage modulus were measured as a function of temperature to identify the temperature at which the poloxamer solutions undergo gelation. Liquid poloxamer solutions were loaded between the 50 mm parallel plates with a gap size of 1 mm. Using an oscillatory strain of 0.2% (determined to be within the LVR for each poloxamer concentration) and an angular oscillation frequency of 6.28 rad/s, liquid poloxamer solutions were heated from 14 to 38 °C with a step size of 1 °C between measurements. Storage modulus of the poloxamer solution was measured after 2-min equilibration times at each temperature. A solvent trap was used to limit solvent evaporation for the duration of the experiment. All studies were performed in triplicate and gelation temperature was determined as the mean temperature at which the storage modulus first surpassed the midpoint between its final (gelled) value and its initial (liquid) value [28]. To quantify changes in poloxamer gelation temperatures due to the addition of diclofenac sodium, gelation temperatures of the resulting solutions were determined using the same procedure.

### 2.3. In vitro Diclofenac Sodium Release Studies

An in-line diffusion cell setup (PermeGear, Hellertown, PA, USA) was used to measure diclofenac release from the poloxamer gels. The membrane mounted into the diffusion cell was a Snakeskin^®^ dialysis membrane with a 10,000 molecular weight cutoff (Thermo Fisher Scientific, Rockford, IL, USA). HEPES buffer with 0.1 mM gentamicin sulfate, pH 7.4, filtered through a 0.22 µm filter, was used as the receiver solution. For all studies, the receiver solution was warmed to 37 °C and maintained at a flow rate of 25 µL/min. Throughout the study, a HAAKE DC1 circulating water bath (Paramus, NJ, USA) was used to warm the diffusion cells to ~32 °C to mimic the approximate clinical application site temperature. Studies were initiated by applying 0.5 mL of 1% *w/v* formulations (5 mg of diclofenac sodium) to dialysis membranes mounted in the diffusion cells, using either a micropipette or syringe. After dose application, cells were occluded for the duration of the experiment. The studied formulations included 1% *w/v* diclofenac sodium in either 17% or 20% *w/w* poloxamer solution; poloxamer formulations were compared to a 1% commercial diclofenac sodium gel (Voltaren^®^, Novartis, Wehr, Germany). Doses of 5 mg were chosen to ensure that adequate drug mass for measurement was collected during the 24-h experiment.

Receiver solution samples were collected every 3 h for 24 h using an automated fraction collector. All studies were performed in triplicate. Diclofenac concentrations in the receiver solution samples were analyzed via high-performance liquid chromatography (HPLC) using the method described below.

### 2.4. In vitro Permeation Studies with Intact and Impaired Skin

Permeation studies were conducted over 24 h using intact porcine ear skin obtained from a local butcher. Porcine ear skin was harvested within hours of sacrifice and stored at −80 °C until use. To ensure a uniform membrane thickness, skin was trimmed to 1 mm thickness using a 75 mm Nouvag AG dermatome (Goldach, Switzerland) and then mounted in the PermeGear in-line cells with the dermal side in contact with the receiver solution. All other conditions were the same as described above for the release studies. Studies were initiated by applying 0.5 mL of 1% *w/v* formulations (5 mg doses) of diclofenac sodium to the membranes (formulations described above). After 24 h, the formulations were removed from the skin surface and the diffusion areas were excised, weighed, and cut into small pieces that were shaken overnight in methanol at 37 °C to extract diclofenac. Diclofenac concentrations in the receiver solution and extracted from skin samples were analyzed via HPLC. All studies were performed in triplicate except for the diclofenac sodium in water formulation, which had 6 replicates.

To test drug permeation through an impaired membrane that would better represent the intended clinical application, the experiment described above using porcine ear skin was repeated but the epidermal surface was subjected to tape stripping to remove the stratum corneum’s barrier potential. During the tape-stripping process, pieces of 3M packaging tape of a size larger than the surface area of the skin section were applied, pressed down via thumb pressure, and quickly removed. Each piece of tape was used for only one application and removal before being discarded. Impairment of the skin barrier was verified via changes in transepidermal water loss (TEWL) measurements made using a Tewameter^®^ TM300 open chamber evaporimeter (Courage+Khazaka Electronic, Köln, Germany). Baseline TEWL values were measured for all skin samples by taking measurements obtained over 10 s after a plateau value was reached. The baseline TEWL measurements were compared to those obtained using the same method after 12, 20, 25, and 30 applications and removals of 3M packaging tape. Tape stripping was concluded when one of the following conditions was met (whichever came first): TEWL values increased by a factor of 5, TEWL values exceeded 60 g/m^2^∙h, or when 30 tape strips were used. Each of these conditions are indications of impaired stratum corneum barrier function [29].

### 2.5. HPLC Quantification of Diclofenac Sodium

Diclofenac concentrations were measured by HPLC using a Shimadzu Prominence *i*-Series LC-2030 Plus (Shimadzu, Columbia, MD, USA) with a Kinetex^®^ (5 µm, 150 × 4.6 mm, C18) column (Phenomenex, Torrance, CA, USA) at a column temperature of 40 °C. The mobile phase consisted of a 40:60 mix of 0.1% *v/v* aqueous formic acid/acetonitrile at a flow rate of 1.0 mL/min (injection volume of 10 µL). Measurements were made at 282 nm with an approximate diclofenac elution time of 3 min.

### 2.6. Data and Statistical Analysis

Release rate constants were calculated using GraphPad Prism 8.1.2 for zero-order kinetics, Higuchi, and Korsmeyer–Peppas release kinetics models according to the equations listed below. The zero-order kinetics and Higuchi models are frequently used to characterize drug release from poloxamer systems, while the Korsmeyer–Peppas model utilizes a power law equation to indicate the mechanism of drug release from a polymeric system [16,30,31,32,33].

Zero-order release equation:

(1) $$Q_{t}\;=\;K_{1}\times \;t\;$$

where *Q_t_* represents the mass of diclofenac sodium that has been released at time *t*, and *K*_1_ represents the zero-order rate constant.

Higuchi release equation:

(2) $$Q_{t}=K_{H}\times \;\sqrt{t}$$

where *Q_t_* represents the mass of diclofenac sodium that has been released at time *t*, and *K_H_* represents the Higuchi dissolution constant. Goodness of fit testing was performed on results from each model.

Korsmeyer–Peppas equation:

(3) $$Q_{t}=\;K_{K-P}\times t^{n}$$

where *Q_t_* represents the mass of diclofenac sodium that has been released at time *t*, *K_K−P_* represents the Korsmeyer–Peppas rate constant, and the exponent n is indicative of the release mechanism. An n of 0.5 indicates that the release fits a Fickian model and *n* > 0.5 indicates a non-Fickian model.

Differences between formulations (percent of dose permeated during 24-h diffusion study and mass of drug present in skin) were assessed using a one-way analysis of variance (ANOVA) followed by Tukey’s multiple comparisons test using GraphPad Prism 8.1.2 (GraphPad Software Inc., San Diego, CA, USA). Changes in TEWL due to tape stripping were analyzed by comparing initial TEWL values to those obtained after tape stripping using a one-tailed paired *t*-test. The level of significance was chosen as *p* ≤ 0.05 for all studies.

## 3. Results

In these studies, we investigated the rheological properties and drug release profiles of poloxamer 407 gels co-formulated with diclofenac sodium and quantified permeation through intact and impaired (tape-stripped) porcine skin.

### 3.1. Determination of Poloxamer Gel Linear Viscoelastic Regions

**Figure 2:**
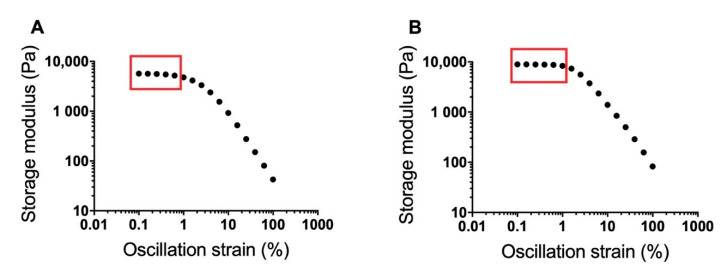
Strain sweeps for 17% (**A**) and 20% *w/w* (**B**) poloxamer gels at 37 °C. Sweeps were conducted over an oscillation strain range from 0.1 to 100%. The approximate linear viscoelastic region of each formulation is boxed. An oscillation strain of 0.2% was chosen from the two linear viscoelastic regions for use in further poloxamer rheology studies.

Strain sweeps of the 17% and 20% *w/w* poloxamer gels (with no diclofenac) conducted via oscillatory rheology were used to determine the LVR of each gel (Figure 2). The storage modulus decreased by ~500 Pa in the range of 0.1 to 0.63% oscillatory strain for the 17% gel and by ~600 Pa in the range of 0.1 to ~1% oscillatory strain for the 20% gel. The storage modulus rapidly decreased for both gels with oscillatory strains beyond these regions, indicating a breakdown in gel structure at higher strains. Based on these data, an oscillation strain of 0.2% was selected for further rheological studies because this was within the LVR of both poloxamer gels.

### 3.2. Effects of Diclofenac Sodium on Gelation

**Figure 3:**
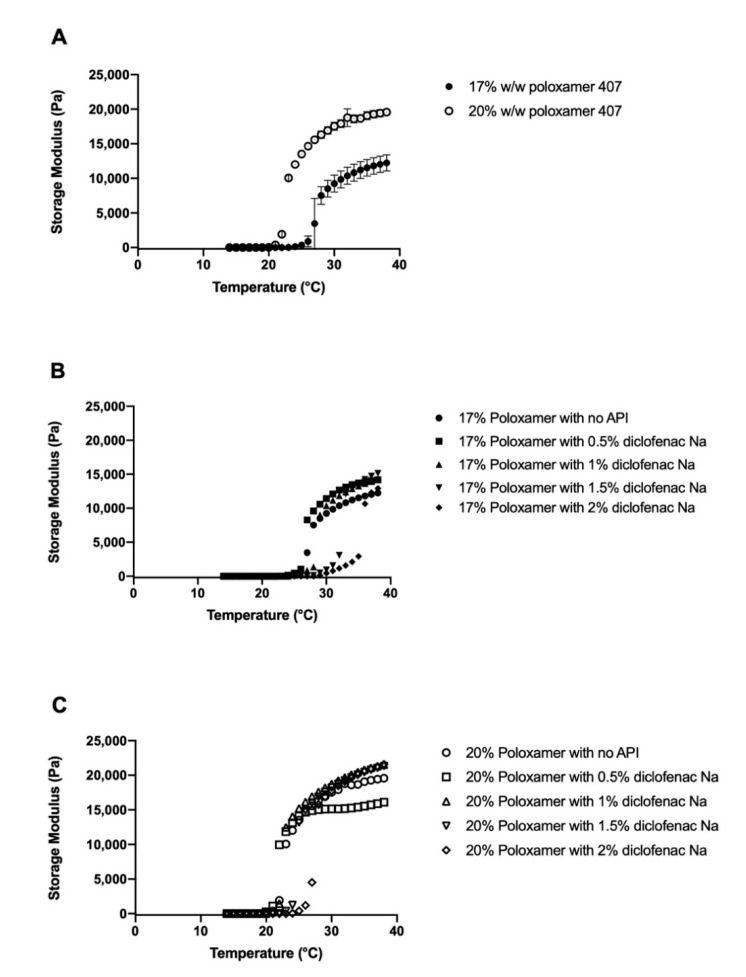
Temperature sweeps from 14 to 38 °C for 17% (filled circles) and 20% (open circles) *w/w* poloxamer 407 solutions without (**A**) and with (**B**,**C**) 0–2% diclofenac sodium. Data are presented as mean ± SD (*n* = 3). SD error bars are omitted for figure clarity in panels (**B**,**C**).

Gelation temperature was defined as the first measured temperature when the storage modulus exceeded the midpoint between the final storage modulus of the gel and the initial storage modulus of the solution [28]. Gelation temperature of the blank poloxamer gels (i.e., no diclofenac loaded) varied inversely with poloxamer concentration (Figure 3A). The gelation temperatures were 27.7 ± 0.6 and 23.0 ± 0.01 °C for the 17% and 20% poloxamer solutions, respectively. Both poloxamer solutions exhibited an increase in storage modulus with heating, and the final storage modulus of the 20% poloxamer solution (19,581.4 ± 497.4 Pa) was higher than the 17% poloxamer solution (12,245.9 ± 1184.0 Pa).

**Figure 4:**
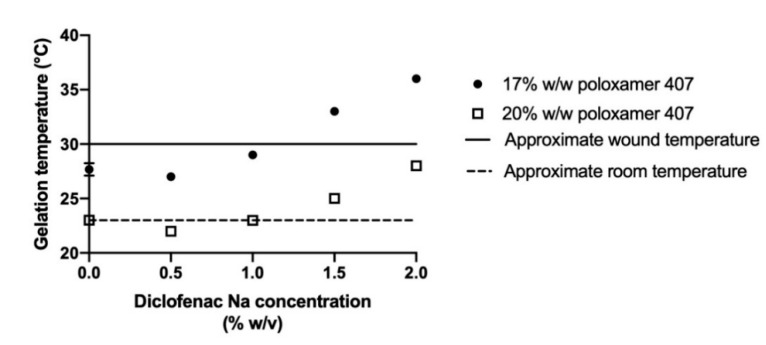
Gelation temperatures for 17% (circles) or 20% *w/w* (open squares) poloxamer 407 solutions containing between 0% and 2% *w/v* diclofenac sodium. The dashed line represents approximate room temperature of 23 °C, which was chosen as the minimum preferred gelation temperature to prevent premature gelation. The solid line represents approximate minimum wound temperature of 30 °C [34]. Data are presented as mean ± SD (*n* = 3). Error bars for most conditions are too small to be seen in the figure.

Gelation temperature generally increased with higher concentrations of diclofenac sodium (Figure 3B,C). The largest changes in gelation temperature were observed with the addition of 2% diclofenac sodium, which correlated to an 8.3 °C increase in gelation temperature for the 17% poloxamer solution and a 5.0 °C increase in the gelation temperature for the 20% poloxamer solution (Figure 4). In order to ensure that the poloxamer formulations would gel at temperatures representative of what would be seen with clinical use, poloxamer solutions containing 1% *w/v* diclofenac sodium were chosen for use in subsequent drug delivery studies.

### 3.3. In vitro Diclofenac Sodium Release and Permeation Studies

**Figure 5:**
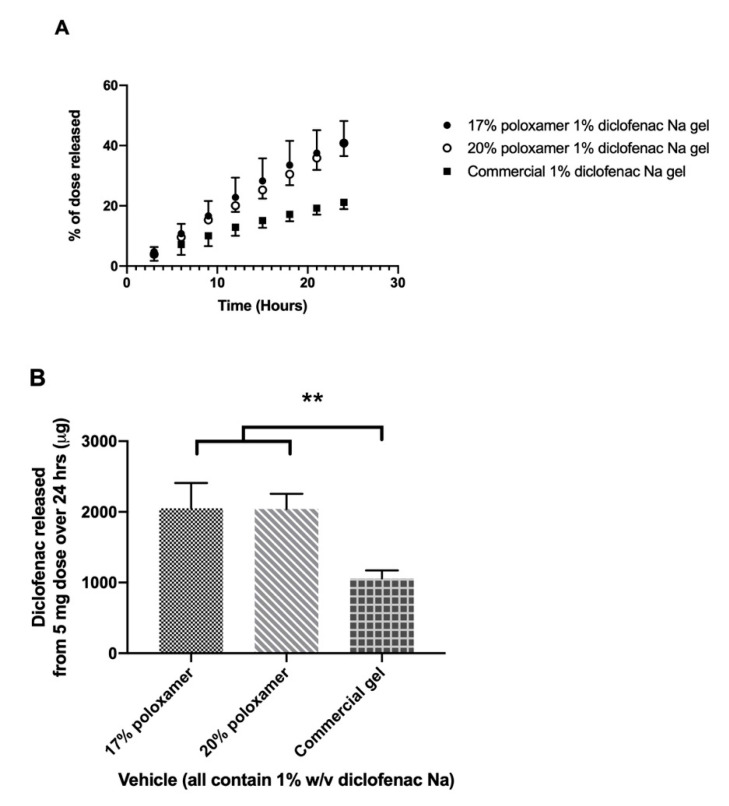
In vitro drug release study using a Snakeskin^®^ dialysis membrane. (**A**) Percent of the initial 5 mg dose released from each formulation over 24 h. (**B**) Total mass of diclofenac sodium released from each formulation over 24 h. Data presented as mean ± SD (*n* = 3). ** *p* ≤ 0.01.

Drug release from the formulated gels was compared to a 1% commercial gel product (Voltaren^®^). Release studies were conducted using a cellulose dialysis membrane which poses little to no barrier to the absorption of drug. The commercially available 1% diclofenac gel released the drug slowly, with 1059.0 ± 111.8 µg released in 24 h. The 17% and 20% poloxamer gels exhibited approximately double the diclofenac sodium release rate, delivering 2051.2 ± 357.8 and 2041.1 ± 215.7 µg over 24 h, respectively (Figure 5).

Drug release kinetics were analyzed by fitting drug release data to the zero-order, Higuchi, and Korsmeyer–Peppas release models (Table 1). The zero-order model fit the release data well for both poloxamer formulations (r^2^ = 0.992 for 17%, r^2^ = 0.998 for 20%), as the drug released vs. time curves were nearly linear. The goodness of fit of the zero-order model was further supported when the Korsmeyer–Peppas model was applied, as the value of n was 1.044 for 17% poloxamer and 1.113 for 20% poloxamer. The release kinetics were not fit by the Higuchi model (r^2^ = 0.833 and 0.799 for 17% and 20% poloxamers, respectively).

**Figure 6:**
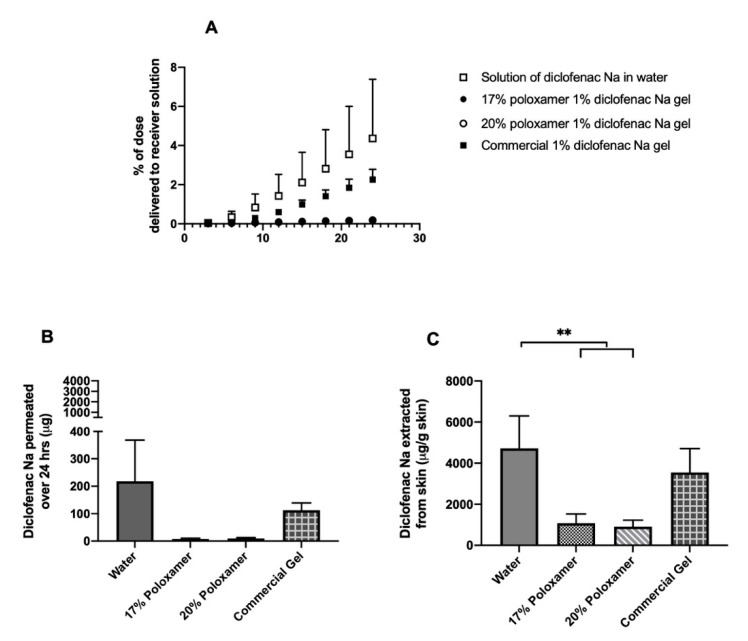
In vitro drug permeation study through intact porcine ear skin. (**A**) Percent of the initial 5 mg dose delivered to the receiver compartment over 24 h. (**B**) Total mass of diclofenac sodium delivered to the receiver compartment over 24 h. (**C**) Total mass of diclofenac sodium extracted per gram of skin upon completion of 24-h permeation study. Data presented as mean ± SD (*n* = 3 for all treatment conditions except solution in water, for which *n* = 6). ** *p* ≤ 0.01.

Permeation studies were conducted using excised porcine ear skin as a representative biological membrane. The rate and extent of diclofenac delivery through intact skin was highest when diclofenac sodium was administered in water, with a mean of 217.9 ± 150.3 µg delivered in 24 h; this condition also presented the largest variability. The rate and extent of diclofenac delivery across the skin and into the receiver solution was lower when administered as a solution in either 17% or 20% *w/w* poloxamer 407, which delivered an average of 7.5 ± 3.1 and 9.3 ± 3.1 µg over 24 h, respectively (Figure 6, Supplemental Table S1). The total amount of diclofenac delivered did not differ significantly between the two poloxamer solutions (*p* > 0.05). The amount of drug that permeated the skin in samples treated with diclofenac sodium in water did not differ significantly from the formulations containing poloxamer, though this is likely a result of the high variability observed with that condition.

An average of 4725.5 ± 1568.8 µg diclofenac per gram of skin was extracted from the intact skin samples treated with diclofenac sodium in water, which was the highest skin amount extracted from all formulations (Figure 6, Supplemental Table S2). Averages of 1079.4 ± 444.4 and 910.9 ± 314.5 µg diclofenac per gram of skin were extracted from the skin samples treated with 17% and 20% *w/w* poloxamer formulations, respectively. An intermediate amount, 3553.3 ± 1156.5 µg diclofenac per gram of skin, was extracted from the intact skin treated with the commercial gel product. Significantly more drug was extracted from the samples treated with diclofenac sodium in water vs. either poloxamer formulation (*p* ≤ 0.05). However, the amount of drug extracted did not differ significantly between samples treated with different poloxamer formulations (*p* > 0.05), between skin treated with the two poloxamer formulations vs. the commercial gel (*p* > 0.05), or between skin treated with diclofenac in water vs. the commercial gel (*p* > 0.05).

### 3.4. Permeation through Tape-Stripped Skin

**Figure 7:**
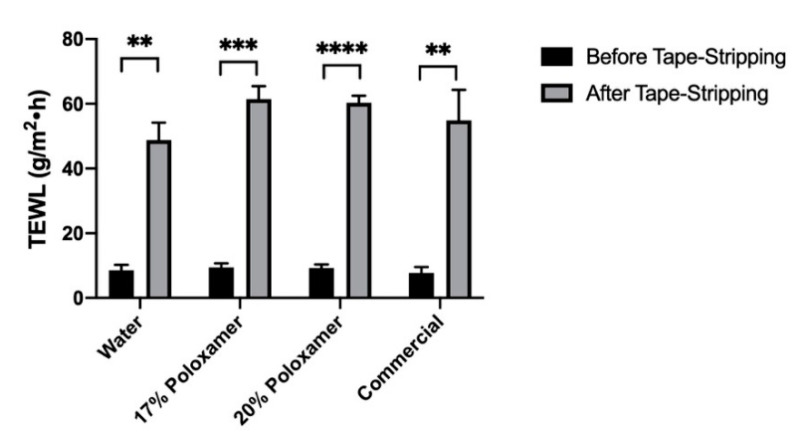
Transepidermal water loss (TEWL) results after tape stripping of skin samples used for the permeation testing in impaired skin samples. The X axis shows what diclofenac formulation was applied to those impaired skin samples. Number of tape strips used varied between samples (described in Methods Section). Data presented as mean ± SD (*n* = 3). ** *p* < 0.01, *** *p* < 0.001, **** *p* < 0.0001.

Impairment of barrier function in excised porcine ear skin was achieved by tape stripping the top layers of the skin. The barrier impairment was quantified using changes in TEWL before and after tape stripping, to ensure relatively comparable levels of impairment. The average TEWL of all studied skin samples increased significantly from 8.7 ± 1.5 at baseline to 56.4 ± 7.3 g/m^2^∙h after tape stripping (the change in TEWL was significant for each skin sample, *p* ≤ 0.05). Average TEWL values before and after tape stripping for the skin samples used with each formulation are shown in Figure 7. This significant increase in passive water loss confirmed that the barrier function of the strata cornea of the skin samples was impaired prior to application of diclofenac sodium formulations.

**Figure 8:**
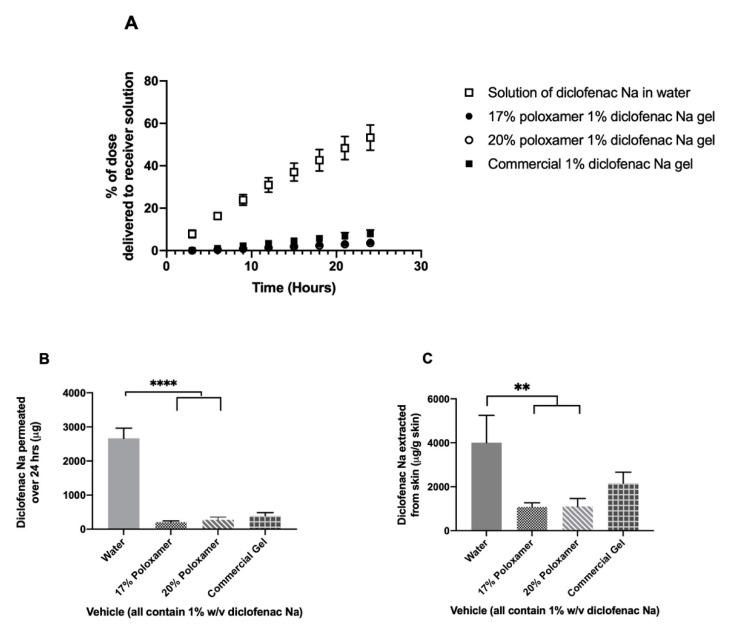
In vitro drug permeation study using porcine ear skin impaired via tape stripping. (**A**) % of the initial 5 mg dose delivered to the receiver compartment over 24 h. (**B**) Total mass of diclofenac sodium delivered to the receiver compartment over 24 h. (**C**) Total mass of diclofenac sodium extracted from skin upon completion of 24-h permeation study. Data presented as mean ± SD (*n* = 3). ** *p* ≤ 0.01, **** *p* ≤ 0.0001.

Diclofenac was delivered most quickly through impaired porcine ear skin when administered in water, with an average mass of 2.66 ± 0.30 mg delivered over 24 h (53.2 ± 6% of administered dose). The 17% or 20% *w/w* poloxamers delivered the drug much more slowly: 204.9 ± 37.5 and 272.8 ± 78.5 µg over 24 h, respectively (*p* ≤ 0.0001, Figure 8, Supplemental Table S1). The extent of diclofenac delivery from the two poloxamer solutions did not differ significantly from each other after 24 h. The commercial gel product delivered an average of 394.6 ± 92.9 µg of diclofenac sodium through impaired skin during the 24-h study, which differed significantly from the solution of drug in water but did not differ significantly from the two poloxamer solutions (*p* > 0.05). Trends for drug content extracted from impaired skin were similar to trends seen for intact skin: highest in the sample treated with a solution in water, from which an average of 4009.4 ± 1243.4 µg diclofenac per gram of skin was extracted (Supplemental Table S2). Significantly less drug per gram of skin was extracted from the impaired skin samples treated with diclofenac sodium in either 17% or 20% poloxamers, from which 1081.6 ± 193.1 and 1108.8 ± 358.0 µg diclofenac per gram of skin were extracted, respectively. An intermediate amount of drug per mass of skin, 2160.5 ± 499.9 µg/g, was extracted from impaired skin treated with the commercial gel product. The mass of drug extracted per gram of skin did not differ significantly between the two poloxamer formulations or between the commercial gel and the poloxamer formulations (*p* > 0.05).

## 4. Discussion

### 4.1. Effect of Diclofenac on Gelation Temperature

For a poloxamer formulation to be clinically useful within a chronic wound, it must form a gel within the wound site. Chronic wound bed temperatures are on average between 30 and 32 °C [34]. Therefore, a poloxamer formulation that gels between 23 (average room temperature) and 30 °C would prevent the product from gelling prior to application or from failing to gel within the wound site. The 17% and 20% poloxamer gels both transition to a gel within this temperature range when no diclofenac is present. While the addition of diclofenac to the poloxamer solutions increased their transition temperature, they were still able to form gels in the relevant temperature range when 1% diclofenac was present in the 17% gels and up to 2% diclofenac was present in the 20% gels (Figure 4).

In order to explain the observed changes in gelation temperature with changes in formulation, it is important to consider the way in which a poloxamer gel forms. Poloxamer solutions transition from a liquid to a gel in a two-step process: micellization followed by gel formation. Given their amphiphilic nature, poloxamers exhibit surfactant-like behavior, supporting their ability to undergo micelle formation. Micelle formation for any surfactant depends on the concentration of molecules in solution and occurs at a concentration called the critical micelle concentration (CMC). Once micelle formation has begun, poloxamer systems form a gel network when the total micellar volume fraction exceeds a critical value [35,36,37]. Since micellization is a necessary step before gelation can occur, any change in the solution composition that affects the poloxamer CMC is expected to have a corresponding effect on the gelation temperature. The clearest example of this relationship for poloxamer molecules is that the CMC decreases as the solution temperature increases [38]. The effect of the decrease in CMC can be observed as the inverse relationship between poloxamer concentration and gelation temperature, as seen in the current studies and in previous research. This relationship exists because solutions of higher poloxamer concentration require a smaller temperature change for the CMC to reach the solution concentration, for micelles to begin forming, and for a gel network to be created.

In our study, the gelation temperature of 17% *w/w* poloxamer solutions increased with increasing diclofenac sodium concentrations of 1% *w/v* and above, and the gelation temperature of 20% *w/w* poloxamer solutions increased with increasing diclofenac sodium concentrations of 1.5% *w/v* and above (Figure 4). These results are in agreement with previous studies, which reported large increases in gelation temperature of mixtures of poloxamers 407 and 188 with the addition of 2.5% diclofenac sodium [18,39]. In another study, addition of anti-inflammatory drugs to poloxamer 407 gels resulted in a small decrease in gelation temperature with concentrations below 0.5% *w/w* of naproxen or indomethacin. This mirrors the decrease in gelation temperature observed in our study with the addition of 0.5% *w/v* diclofenac sodium [40]. The previous study found that the addition of small concentrations of naproxen and indomethacin correlated to a significant decrease in micellar size and aggregation number but an increase in the number of micelles formed at a given temperature, which the authors credited as the reason for the decreased gelation temperature [40]. It is possible that, at the higher diclofenac sodium concentrations used in our study, the decrease in micellar size is enough to mean that a higher temperature is needed for enough micelles to form before the critical volume fraction can be reached for gelation to occur. However, further studies into the effect of diclofenac sodium on micellar size would be needed to verify this hypothesis.

The effect of diclofenac on gelation temperature observed in our study is important for the clinical utility of a poloxamer product for delivery to a chronic wound. At diclofenac sodium concentrations of 1.5% and 2% *w/v*, the 17% poloxamer gelation temperature increased beyond the range of 23 to 30 °C that would be preferred for clinical application (Figure 4). The 20% *w/w* poloxamer solution exhibited smaller increases in gelation temperature with added diclofenac sodium and continued to form a gel within the preferred temperature range when the diclofenac sodium concentration reached 2% *w/v*. In total, five of the studied drug-containing poloxamer solutions gelled within the preferred temperature range for clinical application (Figure 3).

### 4.2. Storage Modulus and Gel Strength

The storage modulus of a viscoelastic material reflects the material’s capacity to store input mechanical energy [41]. The poloxamer solutions exhibited very low storage moduli at temperatures below the gelation point, which is expected given their liquid state (Figure 3). Final storage moduli increased with increasing poloxamer concentration, indicating the formation of stiffer gels (Figure 3). This is consistent with past studies and was expected as a result of an increased number of micelles forming the gel structure [42,43]. The increase in storage modulus with heat creates the potential for a poloxamer delivery system to be administered as a liquid and form a gel at the administration site for prolonged residence time, minimizing the need for frequent re-dosing. Injectable subcutaneous poloxamer vehicles that gel after injection and provide prolonged drug release have been studied in the past; a similar concept would be well suited to deliver a depot dose of an analgesic compound to a chronic wound site with minimal caregiver contact [44,45,46].

The higher storage modulus of the 20% poloxamer formulation at temperatures representative of clinical wounds indicates that it may be better for the intended application, as a stiffer gel is less likely to deform in a wound site due to stresses caused by patient movement. However, although hydrogel storage modulus has been shown in some past studies to correlate with its bioadhesion and fracture strength, the strength of the correlation varies and tends to diminish as the storage modulus increases [47,48,49]. Therefore, further testing of mechanical properties would be helpful in determining whether a 20% *w/w* poloxamer concentration would be more effective than a 17% *w/w* poloxamer concentration in enhancing gel resistance to physical deformation.

Compared to differences in poloxamer concentration, differences in diclofenac sodium concentration had minimal effect on the final storage modulus plateau, which indicates that the poloxamer content of a given gel has a larger effect than diclofenac content on the gel response to input stress. This difference is likely due to partitioning of diclofenac sodium primarily into the interior of the micelles, which limits its influence on the overall gel structure. However, diclofenac is expected to change the size and shape of the micelles forming the gel, so further studies of its effect on micellar size and shape as well as on mechanical properties will be helpful in determining the drug’s effect on overall gel performance [40].

### 4.3. Diclofenac Release from Poloxamer Gels

To measure the effect of poloxamer concentration on drug delivery, 17% and 20% poloxamer formulations were loaded with 1% diclofenac sodium and subjected to release and permeation studies. Diclofenac release from the poloxamer gels was quantified via diffusion studies across a Snakeskin^®^ cellulose dialysis membrane, which poses minimal to no barrier to small molecule diffusion. Under the assumptions that (1) the membrane pore size (MW cutoff 10,000 Da) was sufficiently large compared to the drug molecule size (MW 318.1 Da) to not pass through the membrane and (2) the drug did not interact with cellulose, it is reasonable to equate the relative rate of drug delivery through the membrane to the relative rate of drug released from the formulation [13].

The mass of diclofenac sodium released over 24 h did not differ significantly between the 17% and 20% poloxamer gels (*p* > 0.05), though both gels released significantly more diclofenac than the commercial gel product (*p* ≤ 0.01, Figure 4). The rate-limiting factor for drug release was likely the rate at which diclofenac partitioned out of the poloxamer micelles rather than the rate at which diclofenac diffused through the gel. A past study observed favorable partitioning of diclofenac sodium into micelles of Triton X-100 (a nonionic surfactant-like poloxamer) [50]. It is likely that diclofenac partitions into the hydrophobic cores of poloxamer micelles and partitioning back into the hydrophilic poloxamer tails is disfavored—thus decreasing the available concentration of diclofenac for release. Since poloxamer concentration did not significantly affect diclofenac release, other parameters such as gelation temperature, storage modulus, and ease of handling may be more critical to consider for the intended application.

The release data for diclofenac sodium from poloxamer 407 gels fit best to the zero-order release model and poorly to the Higuchi model, as shown in Table 1. A zero-order drug release model is in agreement with previous studies of poloxamer 407 formulation release kinetics [16,32]. The Korsmeyer–Peppas model gave an n of 1.044 for the 17% poloxamer system and 1.113 for the 20% poloxamer system release data. Since the power law is close to zero order with an n near 1, we can be confident that the data follow non-Fickian kinetics.

### 4.4. Diclofenac Sodium Permeation through Intact Skin

Diffusion studies were performed using excised porcine skin to examine the relationship between diclofenac release and its permeation through a biological membrane. Very little of the 5 mg dose administered in either the 17% or 20% poloxamer solutions permeated through the skin and into the receiver solution during the 24-h study (Figure 6). Additionally, very little of the drug was extracted from the skin after the 24-h study, which indicates that most of the dose remains within the micelles of the poloxamer gel and dissolves within the application site (Figure 6).

The very slow rate of drug release from the poloxamer formulations may explain why significantly less drug was extracted from the skin treated with poloxamer formulations than from skin treated with diclofenac sodium in water. The small amount of diclofenac released would likely have partitioned into the lipid regions of the stratum corneum. The remaining diclofenac in the gel would have remained unavailable to form a skin depot because it was still associated with the micelles, further contributing to the low amount of drug permeating into the receiving solution. Despite the commercial gel showing a lower rate of release in the study using a snakeskin dialysis membrane, there was comparatively more diclofenac observed to pass into the receiver solution or become trapped in the skin from the commercial product vs. the poloxamer gels (Figure 6). The difference in the release and permeation rates of the two formulations is likely because the commercial gel is formulated for delivery to intact skin and includes compounds like propylene glycol and isopropyl alcohol that act as permeation enhancers.

### 4.5. Effect of Skin Impairment on Diclofenac Permeation

Chronic wounds represent a clinical scenario in which the skin has lost its major barrier properties and therefore can no longer adequately prevent absorption of drugs and chemicals from the environment. The primary source of the skin’s barrier function comes from its outermost layer, the stratum corneum. One way of mimicking a chronic wound’s lack of barrier function in vitro is by tape stripping the skin, which removes the stratum corneum and upper layers of the epidermis. Prior literature has reported the use of tape stripping as an effective means of removing the stratum corneum, typically to characterize the layer for its mass and barrier function or to evaluate the percutaneous penetration of topical drugs [51,52,53].

Transepidermal water loss (TEWL) was chosen to measure barrier integrity before and after tape stripping in our studies due to the ease of use and because skin TEWL values increase as barrier integrity decreases, presenting a quantitative way to assess barrier disruption [54,55]. TEWL increased by about five times with tape stripping, proving that the skin barrier was significantly disrupted (Figure 7). This led to a significant increase in the amount of drug permeating from the same formulations through the impaired skin compared to intact skin, which further indicates a reduced barrier function in the tape-stripped skin compared to the intact skin. However, no significant differences in the amount of drug extracted between intact and impaired skin were observed (Figure 8).

The diffusion studies using an impaired porcine ear skin membrane indicated that both 17% or 20% *w/w* poloxamer 407 solutions were effective in limiting the permeation of diclofenac sodium through a membrane with a significantly impaired stratum corneum barrier. Similar rates of diclofenac sodium diffusion through the impaired membrane were observed from poloxamer 407 as from a commercially available topical gel product (Figure 8), indicating that the poloxamer formulation was effective in holding the drug between the wound site and the gelled poloxamer solution throughout the 24-h study. Since diclofenac sodium is intended to act within the wound site itself, reducing inflammation via inhibition of the cyclooxygenase-2 enzyme, maintenance of the drug within the wound site with minimum permeation into the receiver solution is expected to show a therapeutic effect with minimal systemic exposure for the patient [56]. These results therefore suggest that poloxamer 407 may be a useful formulation component in the preparation of a vehicle for clinical delivery of an analgesic compound to a chronic wound site.

## 5. Future Directions

In the current work, we characterized properties of diclofenac-loaded poloxamer gels to make a preliminary determination as to the suitability for a chronic wound application. However, we did not perform any cell culture or animal studies to determine biocompatibility or efficacy, as that was beyond the scope of this project. Ongoing work in cell culture (fibroblasts or keratinocytes) will be needed to test for biocompatibility and possible toxicity of the diclofenac-loaded poloxamer gels. In addition, animal studies will be required to correlate the permeability results with in vivo efficacy (analgesia).

## 6. Conclusions

Optimization of topical products that provide local analgesia and are easy to administer is an important step in the improvement of clinical care strategies for chronic wounds. The results of our work indicate that poloxamer 407 could be useful in a product for this purpose, specifically for delivery of diclofenac sodium. Administration of diclofenac sodium in a poloxamer gel displayed prolonged delivery through both intact and impaired porcine ear skin by slowing the release of the drug when gelled at temperatures that would be expected in a wound site. A diclofenac sodium-loaded poloxamer 407 gel is therefore a promising candidate for further development as a product that could be administered to a chronic wound site with minimal contact to form a gelled in situ depot of an analgesic compound.

## Figures and Tables

**Table 1:** Goodness of fit for each poloxamer system after fitting drug release data to three kinetic models commonly used to study drug release from poloxamer formulations.

Model Parameters	17% Poloxamer			20% Poloxamer		
	Zero-Order	Higuchi	Korsmeyer–Peppas	Zero-Order	Higuchi	Korsmeyer–Peppas
r^2^	0.992	0.833	0.995	0.998	0.799	0.996
k	89.9 µg/h	365.0 µg/h	80.7 µg/h	84.7 µg/h	341.5 µg/h	62.0 µg/h
n (Korsmeyer–Peppas)			1.044			1.113

## Supplementary Materials

The following are available online at https://www.mdpi.com/1999-4923/12/12/1214/s1, Table S1: Mass (µg) of diclofenac sodium delivered to receiver solution from each formulation throughout 24-h drug delivery studies; and Table S2: Mass (µg) of diclofenac sodium extracted per mass (g) of skin treated with each formulation after 24-h drug delivery studies.
