# Supplementary material for: Rheological and Drug Delivery Characteristics of Poloxamer-Based Diclofenac Sodium Formulations for Chronic Wound Site Analgesia
Source: Pharmaceutics. 2020 Dec 15;12(12):1214. doi: 10.3390/pharmaceutics12121214 (PMC7765230; doi:10.3390/pharmaceutics12121214)
Supplement: Supplementary file 1 [file pharmaceutics-12-01214-s001.pdf]

pharmaceutics

Supplementary Materials: Rheological and Drug Delivery Characteristics of Poloxamer-Based Diclofenac Sodium Formulations for Chronic Wound Site Analgesia

Jackson Russo, Jennifer Fiegel and Nicole K. Brogden

Table S1. Mass (μg) of diclofenac sodium delivered to receiver solution from each formulation throughout 24-h drug delivery studies.

	Formulation			
**Membrane**	**Water**	**17% Poloxamer**	20% Poloxamer	Commercial
Dialysis membrane	N/A	2051.2 ± 357.8	2041.1 ± 215.7	1059 ± 111.8
Intact porcine ear skin	217.9 ± 150.3	7.5 ± 3.1	9.3 ± 3.1	112.9 ± 26.3
Tape-stripped porcine ear skin	2664.8 ± 295.7	204.9 ± 37.5	272.8 ± 78.5	394.6 ± 92.9

Table S2. Mass (μg) of diclofenac sodium extracted per mass (g) of skin treated with each formulation after 24-h drug delivery studies.

	Formulation			
**Membrane**	**Water**	**17% Poloxamer**	20% Poloxamer	Commercial
Intact porcine ear skin	4725.5 ± 1568.8	1079.4 ± 444.4	910.9 ± 314.5	3553.3 ± 1156.5
Impaired porcine ear skin	4009.4 ± 1243.4	1086.1 ± 193.1	1108.8 ± 358.0	2160.5 ± 499.9
